# A Ramsey’s Method With Pulsed Neutrons for a T-Violation Experiment

**DOI:** 10.6028/jres.110.074

**Published:** 2005-08-01

**Authors:** Y. Masuda, T. Ino, S. Muto, V. Skoy

**Affiliations:** High Energy Accelerator Research Organization, 1-1 Oho, Tsukuba Ibaraki305-0801, Japan; Joint Institute for Nuclear Research, 141980 Dubna, Moscow Region, Russia

**Keywords:** polarized neutron, symmetry violation, NMR

## Abstract

A Ramsey’s method with pulsed neutrons is discussed for neutron spin manipulation in a time reversal (*T*) symmetry violation experiment. The neutron spin (*s*_n_) is aligned to the direction of a vector product of the nuclear spin (***I***) and the neutron momentum (***k***_n_) for the measurement of a *T*-odd correlation term, which is represented as *s*_n_ · (***k***_n_ × ***I***), during propagation through a polarized nuclear target. The phase control and amplitude modulation of separated oscillatory fields are discussed for the measurement of the *T*-odd correlation term.

## 1. Introduction

**Fig. 1:**
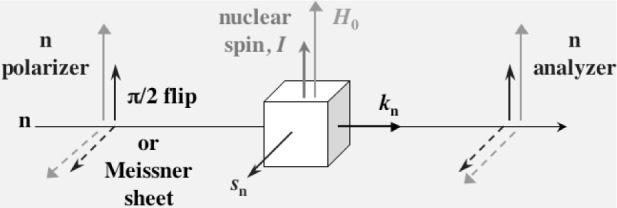
Neutron transmission experiment for the measurement of the *T*-odd correlation term.

A time reversal (*T*) symmetry violation has been intensively studied on polarized neutron transmission through a polarized nuclear target, since very large enhancement is expected in a *T*-odd term, which change sign under *T* [1,2,3]. The *T*-odd term is an angular correlation between the neutron spin (*s*_n_), the neutron momentum (***k***_n_) and the nuclear spin (***I***), which is represented as *s*_n_ · (***k***_n_ × ***I***). For the measurement of the *T*-odd term, the neutron spin and the nuclear spin must be polarized. At the polarization, the neutron and nuclear polarizations are aligned in magnetic fields, while the neutron polarization must be aligned vertical to the nuclear polarization for the measurement of the *T*-odd term. Therefore, we need a magnetic field separator, for example a superconducting sheet between two vertical magnetic fields for the neutron and the nuclear polarizations [4], or we need to rotate the neutron polarization by π/2 before transmission through a polarized nuclear target as it is shown in Fig. 1. Here, we discuss a Ramsey’s method [5] for the neutron spin alignment vertical to the nuclear spin.

## 2. Ramsey’s Method

**Fig. 2:**
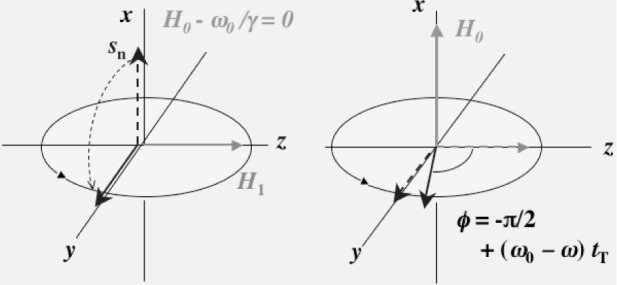
Neutron spin rotation in an oscillatory field and a static field. In a rotating frame of the resonance frequency *ω*_0_, the neutron spin sees only an *H*_1_ field and then rotates by π/2 under the condition *γH*_1_*t*_r_ = π/2. After the rotation, the *H*_1_ field is switched off and then the neutron spin rotates about the static field.

**Fig. 3:**
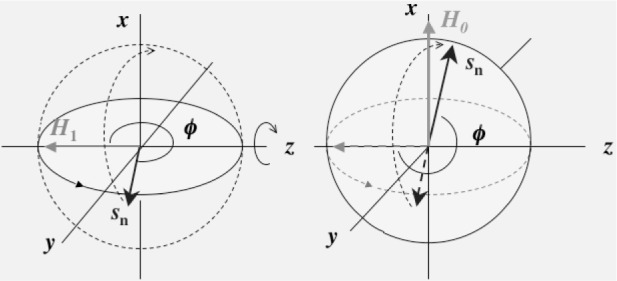
Neutron spin rotation in the second oscillatory field. A plane where the neutron spin rotates under the static field rotates by π/2 under the second *H*_1_ field.

The Ramsey’s method uses two separated oscillatory fields. The oscillating fields 2*H*_1_cos*ωt* are vertical to the static fields *H*_0_ which hold the neutron spin. Here, the frequency of an oscillating field is denoted as *ω*. The oscillating field is represented as a sum of clockwise and counterclockwise rotating fields, *H*_1_(exp(*iωt*) + exp(−*iωt*)). In a rotating frame of a frequency *ω*, an effective magnetic field on the neutron spin is represented as $H_{0}-\tilde{\omega }/\gamma +H_{1}$. At *ω*=*ω*_0_, the effective field becomes ***H***_1_ and then the neutron spin rotates around ***H***_1_. Here, *ω*_0_ = *γH*_0_. The oscillatory field is applied to the neutron spin for a time interval of *t*_r_ so that the neutron spin becomes vertical to a static field, namely the equation *γH*_1_*t*_r_ = π/2 is satisfied as it is shown in the left hand side of Fig. 2. After the π/2 rotation, the neutron spin rotates around the static field for a time interval of *t*_T_ as it is shown in the right hand side of Fig. 2. The phase difference between the neutron spin rotation and the rotating field is represented as *ϕ* = −π/2 + (*ω*_0_ − *ω*)*t*_T_. Next, the second oscillatory field, which is coherent with the first oscillatory field, is applied. In the second oscillatory field, the neutron spin rotates back to the direction of another static field as it is shown in Fig. 3. After the rotation, the angle of the neutron spin with the static field direction becomes *ϕ* − 3π/2. The projection component of the neutron polarization *P*_n_ on the static field becomes

$$P_{R}=P_{n}\cos[(\omega_{0}-\omega )t_{T}],$$ (1)

which is held by the magnetic field and then analyzed.

## 3. Spin Manipulation for the Measurement of the *T*-Odd Term

**Fig. 4:**
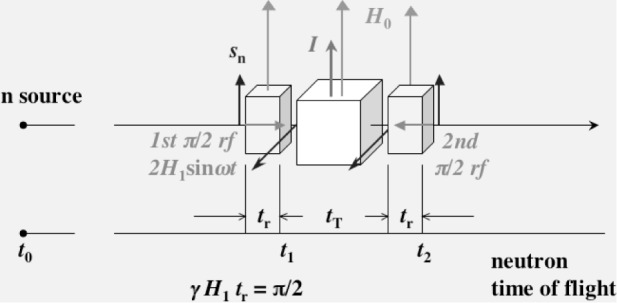
Neutron spin manipulation by Ramsey’s method for the measurement of the *T*-odd correlation term. When the neutron exits from the first π/2 coils, the phase of the oscillatory field (rf field) is adjusted to be π/2 so that the neutron spin is aligned in the direction of ***k***_n_ × ***I***. Here, the phase defined as 0 at the direction of ***k***_n_ × ***I***. The phase of the second oscillatory field is adjusted to be −π/2 so that the neutron spin is rotated back to the static field.

In Fig. 4, neutron spin manipulation for the measurement of the *T*-odd term is shown. The neutron spin is rotated in the first π/2 coil. The neutron spin is vertical to the rotating *H*_1_ field upon the rotation. After the π/2 rotation, the neutron spin is placed in the plane vertical to the static field. Therefore, the neutron spin direction in the vertical plane depends upon the phase of the *H*_1_ field. The *H*_1_ phase must be controlled for the neutron spin alignment to the direction of ***k***_n_ × ***I*** for the measurement of the *T*-odd term. In the polarized nuclear target, Larmor precession must be cancelled by pseudo-magnetic precession [6] to keep the *T*-odd correlation *s*_n_ · (***k***_n_ × ***I***). After transmission through the polarized nuclear target, the neutron spin is rotated back to the static field, and then the projection component of the neutron polarization on the static field *P*_n_cos(*ωt*_T_) is analyzed. As a result, the following conditions should be satisfied for the radio frequency (rf) fields.

1. When the neutron exits from the first and second π/2 coils, the phase of the oscillatory field (rf field) should be π/2 and −π/2, respectively, which are the angles with the direction of ***k***_n_ × ***I***.
2. The value of *ωt*_T_ should be 2nπ in order to obtain the largest effect.

A pulsed neutron beam from a spallation neutron source can satisfy these requirements. A typical neutron pulse width of the spallation source is *δt* = 1 µs. The variation of rf phase in the neutron pulse is *ωδt*. The magnetic field strength in the nuclear spin polarizer is a key parameter. Lower magnetic field is preferable to reduce the error of the rf phase. The nuclear spin of ^131^Xe can be polarized at a low field, for example at 0.5 mT by means of a rubidium spin exchange optical pumping. The neutron Larmor frequency is 15 kHz at 0.5 mT, therefore, the variation of the rf phase in the neutron pulse becomes

$$\omega \delta t=15\times 10^{3}\times 2ð\times 1\times 10^{-6}=0.09.$$ (2)

The accuracy of the neutron spin alignment with ***k***_n_ × ***I*** is limited by the timing signal of the neutron pulse. If we use the smaller magnetic field at the π/2 coil, the uncertainty of the rf phase will be further reduced since we can use the lower rf frequency.

The ^131^Xe nucleus has a *p*-wave resonance at a neutron energy of 3 eV with a resonance width of about 100 meV. The *T*-odd effect is largely enhanced in the *p*-wave resonance, therefore the measurement will be carried out in the resonance. If we place the polarized Xe target at 20 m from the neutron source, the resonance width corresponds to a neutron time of flight (TOF) width of 14 µs, and then the rf phase varies by

$$\omega \delta t=15\times 10^{3}\times 2ð\times 14\times 10^{-6}=1.3$$ (3)

in the resonance. However, the variation can be analyzed by the TOF measurement and then the error of the rf phase will be reduced to the same order of error which arises from the neutron pulse timing.

The neutron TOF at 3eV for the length of 20 cm between the two π/2 coils is about 10 µs. However, the phase difference which arises from the neutron TOF will be compensated by the phase difference between the two separated oscillatory fields.

## 4. Development of Pulsed Neutron Ramsey’s Method

**Fig. 5:**
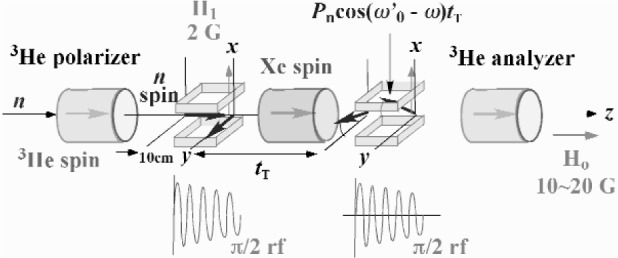
Application of Ramsey’s method to pseudomagnetism. The neutron beam is longitudinally polarized upon transmission through a polarized ^3^He filter. The neutron polarization is rotated from the longitudinal to transverse direction. The transverse neutron polarization rotates about the longitudinal magnetic field and the pseudomagnetic field of polarized Xe. After the precession, the neutron polarization is rotated back to the longitudinal direction and then analyzed by another polarized ^3^He filter.

The Ramsey’s method can be also applied to the measurement of pseudomagnetism. For the cancellation of the Larmor precession with the pseudomagnetic precession, we need to know the value of the pseudomagnetic field. The value is expected to be 0.5 mT at a ^131^Xe polarization of 50 % and a ^131^Xe pressure of 2 bar [7]. The accuracy of the cancellation is limited by the error of the pseudomagnetism. We are developing a pulsed neutron Ramsey’s method, which is shown in Fig. 5. The neutron beam is longitudinally polarized upon passing through a polarized ^3^He filter. Neutron spins are rotated by π/2 from the longitudinal to transverse direction in the first π/2 coil. Xe spins are also polarized in the longitudinal direction. The neutron spins rotate around the neutron beam axis under magnetic and pseudomagnetic fields. The rotation is represented as a phase *ω*_0_*t*_T_. After transmission through the polarized Xe target, the neutron spins rotate back to the longitudinal direction. The projection component on the magnetic field, which is written in Eq. (1), is analyzed by the second polarized ^3^He filter. The two rf fields are synchronized with the neutron pulse and their amplitudes are modulated. The amplitude modulation is proportional to 1/*t*_TOF_ so that the π/2 rotation is satisfied for different neutron energies. We can obtain the value of the pseudomagnetic field from the variation of the phase shift *ω*_0_*t*_T_, which is observed when the Xe polarization is switched on.

The ^3^He polarization is a key parameter. We developed the ^3^He polarization by a rubidium spin exchange optical pumping in a birefringence cell, a sapphire cell [8]. The ^3^He polarization was 63 % at a pressure of 3.1 bar in a cell of 3 cm inner diameter and 4.7 cm length. The neutron polarization by the ^3^He cell was higher than 90 % at thermal and cold neutron energies. We can apply the ^3^He cell to the pseudomagnetism measurement at these neutron energies. We also developed the Xe polarization by the optical pumping. The Xe polarization was measured by a pulsed neutron transmission. A preliminary value of ^131^Xe polarization was 20 % [9]. The value is enough to measure the pseudomagnetism.
